# Indirect questioning method reveals hidden support for female genital cutting in South Central Ethiopia

**DOI:** 10.1371/journal.pone.0193985

**Published:** 2018-05-02

**Authors:** Mhairi A. Gibson, Eshetu Gurmu, Beatriz Cobo, María M. Rueda, Isabel M. Scott

**Affiliations:** 1 Department of Archaeology and Anthropology, University of Bristol, Bristol, United Kingdom; 2 Centre for Population Studies & Institute of Development and Policy Research, Addis Ababa University, Addis Ababa, Ethiopia; 3 Department of Statistics and Operational Research, Science Faculty, University of Granada, Granada, Spain; University of Exeter, UNITED KINGDOM

## Abstract

Female genital cutting (FGC) has major implications for women’s physical, sexual and psychological health, and eliminating the practice is a key target for public health policy-makers. To date one of the main barriers to achieving this has been an inability to infer privately-held views on FGC within communities where it is prevalent. As a sensitive (and often illegal) topic, people are anticipated to hide their true support for the practice when questioned directly. Here we use an indirect questioning method (unmatched count technique) to identify hidden support for FGC in a rural South Central Ethiopian community where the practice is common, but thought to be in decline. Employing a socio-demographic household survey of 1620 Arsi Oromo adults, which incorporated both direct and indirect direct response (unmatched count) techniques we compare directly-stated versus privately-held views in support of FGC, and individual variation in responses by age, gender and education and target female (daughters versus daughters-in-law). Both genders express low support for FGC when questioned directly, while indirect methods reveal substantially higher acceptance (of cutting both daughters and daughters-in-law). Educated adults (those who have attended school) are privately more supportive of the practice than they are prepared to admit openly to an interviewer, indicating that education may heighten secrecy rather than decrease support for FGC. Older individuals hold the strongest views in favour of FGC (particularly educated older males), but they are also more inclined to conceal their support for FGC when questioned directly. As these elders represent the most influential members of society, their hidden support for FGC may constitute a pivotal barrier to eliminating the practice in this community. Our results demonstrate the great potential for indirect questioning methods to advance knowledge and inform policy on culturally-sensitive topics like FGC; providing more reliable data and improving understanding of the “true” drivers of FGC.

## Introduction

Over 200 million of the world’s female population currently live with female genital cutting (FGC), which involves the removal of, or injury to, their genital organs for non-medical reasons [1]. Twenty years since the first joint WHO/UN statement against FGC, elimination of the practice remains a key unmet development goal (SDGs, Target 5). The health implications, particularly from the more severe types of FGC, are well-documented and include infection, obstetric, psychological and sexual problems [2]. However, high quality data on FGC behaviour and norms, which are essential to the design of effective intervention programmes, remain elusive. The practice occurs in private and the effects are not externally visible. Furthermore it is a sensitive topic, making people inclined to hide their “true” views on the topic. For example, people may feel pressure to understate the prevalence of the practice or their preference for it (due to its illegality) [3], or to overstate it (due to social pressures, e.g. as a requirement of marriage). The possibility of both understatement and overstatement makes it especially difficult to assess to the prevalence and predictors of support for FGC at present.

To date most studies exploring FGC behaviour have relied on self-report data derived from direct questioning methods, with many indicating that rates of (and interest in) FGC are broadly in decline [4]. For example, the direct questions used in the Ethiopian Demographic and Health Surveys have revealed that FGC prevalence in Ethiopia declined from 80% in 2000 to 74% in 2005, and directly-stated support for the practice almost halved (60% to 31%) over the same 5 year period [5]. FGC status obtained through physical examination (the gold standard for FGC studies) rarely exists to substantiate these claims, and where it does, has revealed discordance between the two measures (indicating both over-reporting and under-reporting) [6, 7]. This disparity, between clinical and self-report data, confirms that people may be inclined to conceal FGC behaviour (and their support for it) in surveys. Yet, physical examination is intrusive and expensive (requiring a health professional), and thus is infeasible as a tool to guide research and policy.

To address these issues we employ an “indirect” questioning method (unmatched count technique or UCT, see Methods) to explore variation in support for FGC within one rural Ethiopian community where FGC is illegal. These kinds of indirect questioning methods can anonymously obtain responses to sensitive questions [8–10], and can be used to gauge the extent and direction of ‘true’ responses, as well as individual variation in hidden views or behaviours. These methods permit the estimation of population-level support for cutting without revealing the individual preferences to the interviewer. Further, by comparing indirect question responses with those from traditional direct questioning, it possible to identify the extent to which behaviours (or views in support of the practice) are concealed (over or under-reported) using direct questioning. These techniques have recently led to an improved understanding of civic issues such as racial prejudice and poaching [11, 12], but have been relatively under-applied to substantive health issues in low income settings. There is, however, growing interest in the potential of UCT to accurately record sensitive reproductive health-related behaviours, for example, improving abortion statistics [13]. One recent study has used them to explore women’s views on female genital cutting among the Afar (a pastoralist community located in the North of Ethiopia: [14]). While ours not the first UCT study to uncover hidden support for FGC, we suggest improvements to study design (e.g. we consider the views of men, as well as women), and develop the UCT data collection methods to make them applicable in low income settings, something missing from the previous study. These are discussed in further details in the Methods section below.

How and why FGC is maintained in some populations despite the health consequences for women and efforts to eliminate the practice has been of long-standing interest for social and medical anthropologists [15]. Recently evolutionary anthropologists have also addressed the question and are providing important and novel insights which help to explain variation in FGC behaviour (and acceptance of it) [16, 17]. For some, the evolutionary origin and persistence of the practice has been linked to controlling women’s sexual desires and behaviours before or within marriage, which increases male paternity certainty [18]. In other words, it lessens the chance of pre-marital or extra-marital affairs, and eliminates the risk to men of raising unrelated offspring (rather than their own genetically related progeny). To date, however, these ideas remain largely untested using empirical data. Here, based on a similar evolutionary perspective, and drawing on evolutionary kin selection and sexual conflict theories, we explore the extent to which relatedness is important in explaining individual variation in views in support of the practice. One prediction is that there is likely to be more support to cut daughters-in-law than there is to cut daughters. This is based on the assumption that the adverse health consequences of FGC in closely related kin (e.g. daughters), may be of greater concern than non-biological kin (e.g. daughters-in-law); while paternity certainty (and mate-guarding) may be of greater concern when relating to daughters-in-law than to daughters. An alternative proposal derived from evolutionary theory is that cutting will be endorsed equally for both daughters and daughters-in-law, as parents interests in both are closely tied [19]. Any health risk to either groups of women from the procedure may impact on parents’ reproductive fitness (lead to fewer surviving grand-offspring). Further any benefits of cutting may be equivalent too. For example, if cutting signals sexual fidelity, daughters who are cut may have better marriage prospects, and receive greater support from their in-laws.

In this study we combine direct and indirect questioning methods to explore concealed support for FGC according to individual circumstances including gender, age and level of education of the respondent, as well as the characteristics of the target female. Individual variation in level of support for FGC based on gender, age and education are well known in the anthropological literature [20–22]; however the reported effects vary due to contextual differences between populations (as well as differences in methodologies). The extent to which the desirability of FGC varies between categories of female kin (daughters and daughters-in law) has to our knowledge, not previously been tested. Our data are drawn from a rural Ethiopian Arsi Oromo community where household surveys over a five year period have revealed a recent and rapid decline in self-reported FGC prevalence rates (from 90% in 2010 to <20% in 2015). This sudden drop in reporting rates is an indication that women in this community have become more inclined to conceal their FGC status.

## Methods

In 2016 a socio-demographic household survey was undertaken with 1620 adults living in rural sub-districts of Arsi and East Shewa zones, Southern Oromia. This included an equal and randomly selected sample of adult (> = 18 years) male and female respondents, married and unmarried respondents (one of each sex, and one of each marital status from alternate households selected from a village plan, and household member lists provided by the district office). The survey was undertaken in the respondent’s house (or within their compound) by a trained same-gender interviewer fluent in the local language, Oromiffa. No other adult was present. Prior to the main survey, focus group discussions were undertaken to develop the questionnaire (e.g. choosing the items included in the unmatched count technique list), the survey was then piloted in a neighbouring village, and all interviewers received training in the survey protocols [see S1 Fig and S2 Fig]. Research and Ethical approval to undertake this study was granted by the Ethics Committees at the University of Addis Ababa and the University of Bristol. Informed written consent (or fingerprint consent) was obtained from each participant in the study.

To compare openly-declared and privately-held support for FGC, the survey employed direct questions on the desirability of FGC, as well as the unmatched count technique (UCT), an indirect questioning method designed to mitigate the problems associated with sensitive survey topics. UCT is sometimes referred to at the List Experiment or the Item Count Technique [9, 11]. All respondents were asked about the desirability of FGC for both a hypothetical daughter, and a prospective daughter-in-law. Details of the questions posed in each survey version can be found in S1 Fig, and details of the sampling methods are outlined in S2 Fig.

**Fig 1:**
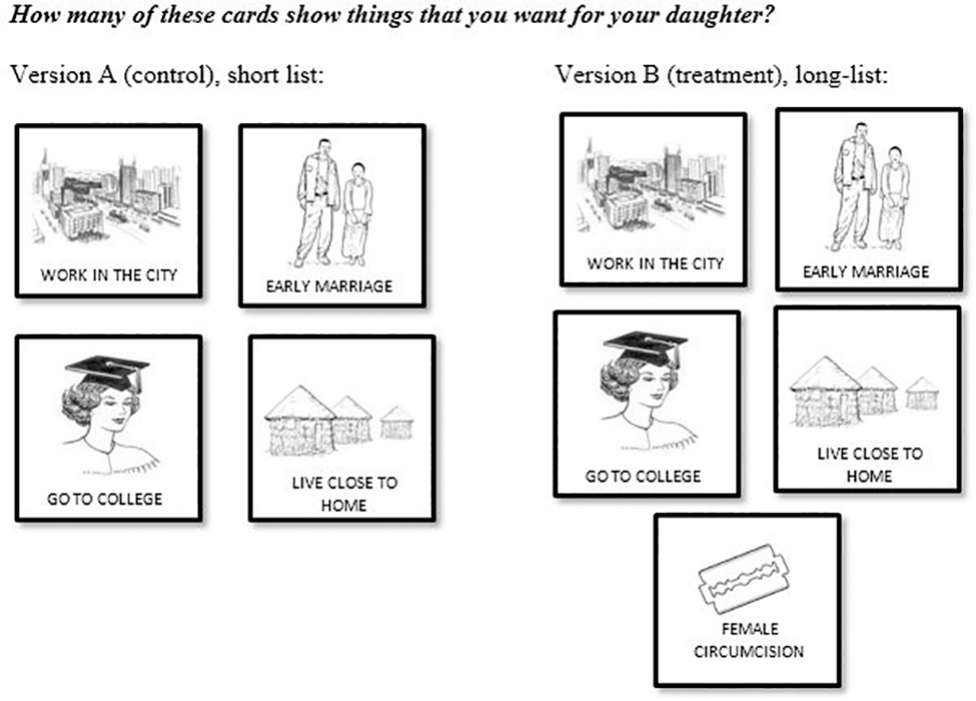
Unmatched count techniques (UCT) question materials. UCT items were presented on illustrated cards, to facilitate comprehension and randomize item presentation order. (S1 Fig includes full details of questions).

There were four different versions of the survey which were randomly assigned to respondents, these included direct and indirect questions (Version 1 and 2), a control and treatment condition (lists with and without the sensitive item, FGC; Version A and B). Seventy percent of the sample undertook a survey with the indirect UCT question (n = 1112), with participants equally and randomly assigned to either a control or a treatment condition (see Fig 1). Individuals in the control condition (Version 2A) were shown cards with four (non-sensitive) items and asked how many (but not which particular) items are desirable for their daughter or daughters-in-law, while the treatment group (Version 2B) was shown the same set of cards but with the sensitive item added (the five item treatment) [S2 Fig on how UCT item lists were generated, and tested]. The difference between the mean number of items reported in the two conditions provided an estimate of those in favour of cutting (the sensitive item) for the entire population.

The remaining 30% of the sample answered direct questions (n = 508), and were also randomly assigned to either a four item control group (Version 1A) or five item treatment group which included the additional FGC card (Version 1B). In this case respondents were asked to identify whether each of the items on the cards were desirable for a hypothetical daughter or daughter-in-law. The proportion of individuals saying “yes” to the FGC card in the five card treatment group (n = 331) provided an estimate of directly expressed “popularity” of FGC. The proportion of “yeses” for the four non-FGC cards was compared across control (1A) and treatment (1B) groups to check that adding an FGC item did not influence how people responded to the other four cards (a design effect). This “additional item” test was satisfied [see S2 Fig for more detail on our sampling strategy and data quality tests].

A comparison of the estimates obtained from direct and UCT questioning methods provided a measure of the extent to which privately-held views differed from those openly-stated (i.e. FGC support that was over or under-reported when asked directly). To ensure an accurate comparison could be made between direct and UCT estimates we used a single question/measure of FGC support in both direct and UCT surveys “Would you want [item named] for your daughter (or daughter in law)?” This is an improvement on previous studies which have relied on estimates obtained from non-identical measures of the sensitive item (e.g. different questions/scales used to define support for the sensitive behaviour in DT and UCT surveys; [14]), which has the potential to introduce inaccuracies in comparative analyses.

Another novel aspect of this UCT study design was the use of cards with pictures for each item included in the list, allowing randomized presentation of the list items and improved respondent comprehension. The participants were able to handle the cards as each item was read out by the interviewer, who could shuffle the cards between interviews (see Fig 1). Previous UCT studies have required participants to read the list of items, which are less well suited for use in populations with literacy issues, and present challenges for item randomization. All items included in the list were generated from focus group discussions and picture cards quality tested during piloted phases of the study [See S2 Fig].

One challenge for all indirect questioning methods is in minimizing biases in responses. Biased responses to the sensitive time may occur where informants become cognisant of the nature of the survey and/or feel the anonymity of responses may be compromised. For example, in the pioneering study by De Cao and Lutz [14] the sensitive question was embedded in a survey on a related topic (women’s reproductive health), and the survey was administered by people known to hold a particular viewpoint (sexual health charity workers trying to eliminate the practice). To minimize potential biases in responses: participants in our study answered either direct questions or indirect questions (not both); the survey included no additional questions related to the sensitive topic; and was administered by professional enumerators of the same gender not known to the informants. Further, the items included in the UCT list were carefully chosen to so as to minimize the chance of floor and ceiling effects–that is, of participants preferring either all or none of the items. Such effects can be problematic because they effectively reveal the participant’s attitude to the sensitive item [9, 11]. Following practices advocated in prior research to mitigate floor/ceiling effects, [11] four items were selected such that: a) one item was expected to be unpopular (early marriage) b) one item was expected to be popular (go to college) and c) two items were expected to be seen as incompatible (work in the city, and live close to home).

### Statistical analyses

Analyses were performed using freely available R software, version 3.3.2 (2016-10-31) [23]. To contrast the proportions between the direct question (DQ) method and unmatched count techniques (UCT), and for subgroups (in both DQ and UCT methods) we used a contrast of equal proportions (calculating the value of the statistic and its associated p-value). We also performed multivariate analyses using generalized regression models (with and without iterations of the covariates) developed by [24, 25], and [9] and applied in other UCT analyses including [26, 27] and [14]. These multivariate analyses have not been included in this paper, as none of the tested models fitted well (possibly because some of the sub-groups have a very small sample size (see S1 Table)). This is one important challenge for this methodology: the UCT provides respondents with privacy at the expense of statistical efficiency [25], and large sample sizes are required. S1 Dataset includes the full dataset used to perform these analyses.

### Study population

The Arsi Oromo are agropastoralists who combine cattle rearing with maize, wheat and sorghum cultivation in the rural low-lying areas of Arsi and East Shewa administrative zone, in Oromia, South Central Ethiopia. Family sizes are large, but agricultural land is limited [28]; and off-farm employment opportunities are rare [29]. Schooling is limited: a third of adults in our sample had never attended school, the rest attending for on average less than two years. The community has limited access to media and urban exposure: of our sample, over a third (36%) had never listened to radio, 53% had never watched TV, <20% reported never visiting a big town or city. Inheritance patterns are patrilineal, and wealth inequality is relatively low due to a programme of government land redistribution in the late twentieth century [30]. Arranged marriages are central to alliance formation between un-related families, often involve large cash bridewealth payments which are transferred from groom to the bride’s family, and post-marital residence is predominantly patrilocal (a daughter moves to join their husband’s village and lineage at marriage) [31].

FGC among the Arsi Oromo involves a nick or cut to the clitoris, and is linked closely to marriage. Cutting occurs in adulthood in the months leading up to marriage (typically women marry in their late teens) and is performed in private, by traditional female practitioners. Since 2004 FGC has been illegal in Ethiopia, and this is widely known within the community (98% of our sample were aware that there is a law preventing FGC), although there have been no local incidences of anyone being brought to trial. To date, openly declared rates of FGC from this community suggest that the practice is either in decline, or is being increasingly under-reported. Over a five year period, the number of women directly reporting that they had been cut (in household census surveys) dropped from 90% of women in 2010 to <20% in 2015.

## Results

A total of 1620 adults were included in the survey and analyses, this included equal numbers of males and females (811 males, and 809 females). The non-response rate was zero. Over one third of the sample (n = 581) had never attended school. For those who had received formal education (n = 1039) the majority had completed less than three years in school. For the purposes of analyses, the sample was divided into two age groups: 18–25 years, and 26+ years. The results are summarized in Table 1 below.

**Table 1:** Direct question (DQ) and unmatched count technique (UCT) estimates indicating support for FGC in daughters and daughters-in-law by gender, age and education level of the respondent.

Respondents	Relative	DQ estimate (SE)^a^	UCT estimate (SE)^b^	P-values^c^
**All**	**Daughters**	0.073 (0.014)	0.197 (0.040)	0.003
	**In-laws**	0.082 (0.014)	0.250 (0.043)	<0.001
	**Both**^**d**^	0.077 (0.010)	0.224 (0.030)	<0.001
**Male**	**Daughters**	0.074 (0.020)	0.142 (0.071)	0.356
	**In-laws**	0.092 (0.022)	0.228 (0.070)	0.064
	**Both**	0.083 (0.015)	0.185 (0.050)	0.051
**Female**	**Daughters**	0.071 (0.019)	0.256 (0.044)	<0.001
	**In-laws**	0.071 (0.019)	0.278 (0.053)	<0.001
	**Both**	0.071 (0.013)	0.267 (0.034)	<0.001
**18–25 years**	**Daughters**	0.072 (0.023)	0.094 (0.074)	0.781
	**In-laws**	0.072 (0.023)	0.061 (0.079)	0.896
	**Both**	0.072 (0.017)	0.078 (0.054)	0.923
**26+ years**	**Daughters**	0.073 (0.017)	0.249 (0.047)	<0.001
	**In-laws**	0.086 (0.018)	0.344 (0.052)	<0.001
	**Both**	0.080 (0.012)	0.296 (0.035)	<0.001
**No education**	**Daughters**	0.116 (0.027)	0.155 (0.068)	0.595
	**In-laws**	0.124 (0.028)	0.233 (0.073)	0.159
	**Both**	0.120 (0.019)	0.194 (0.050)	0.165
**Some education**	**Daughters**	0.045 (0.014)	0.219 (0.049)	<0.001
	**In-laws**	0.054 (0.015)	0.257 (0.053)	<0.001
	**Both**	0.050 (0.010)	0.238 (0.036)	<0.001

^a^Derived from direct questions (DQ)

^b^Derived from indirect questions (the unmatched count technique, UCT)

^c^ P-value refers to significance of difference between DQ and UCT estimates.

^d^ Mean estimates derived from responses to two questions regarding daughters and daughters in law.

### Direct versus indirect response methods

**Fig 2:**
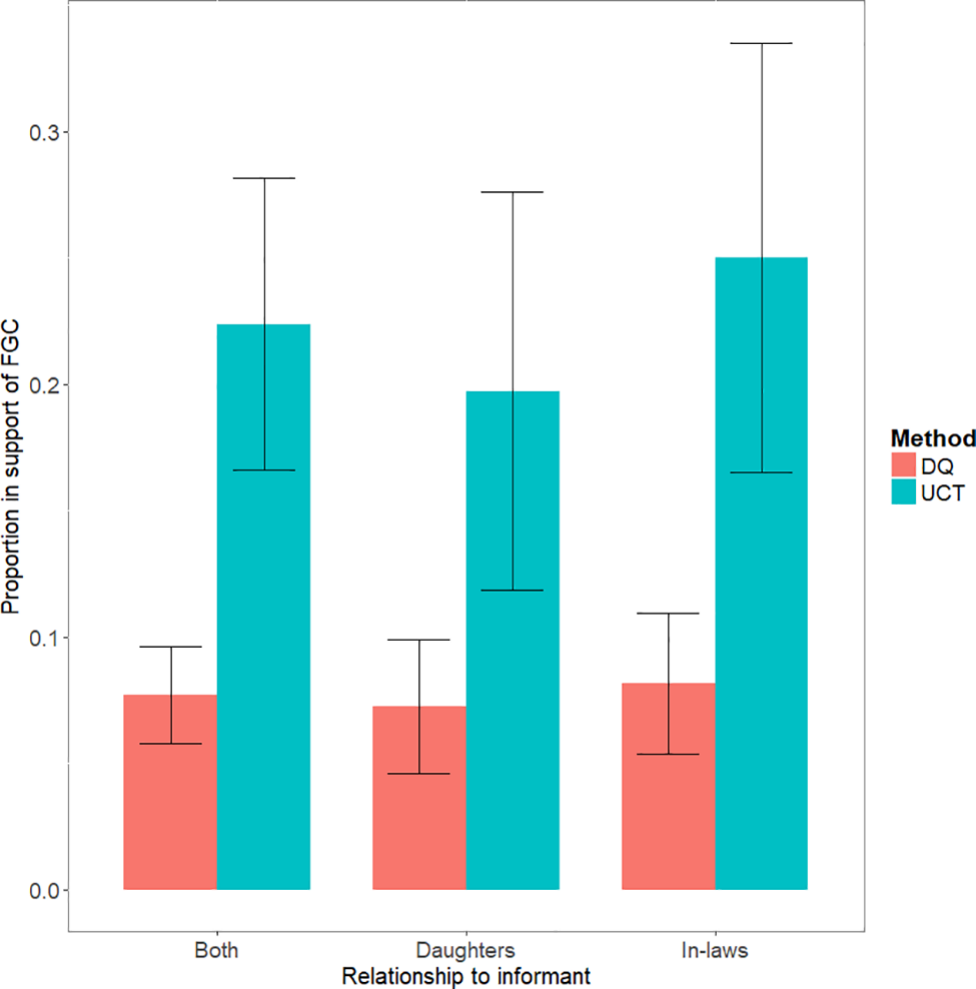
Bar chart comparison of the proportion of people in favour of FGC for a hypothetical daughter or daughter-in-law or both combined, using DQ and UCT responses (estimated proportions) (n = 1620). The error bars represent confidence intervals at 95%.

The indirect response method, unmatched count technique (UCT), revealed that people privately had higher levels of acceptance of FGC behaviour than was admitted openly through direct questioning (DQ). Overall, a low proportion (7.7% (95% CI [5.8–9.6]) of directly posed questions about FGC were met with a positive response (in favour of FGC), whereas UCT indicated that “true” support was three times higher, at approximately 22.4% (95% CI [16.6–28.2]) of responses (difference: p<0.001, see Table 1 and Fig 2).

### Kinship relationship to women (daughter versus daughter in law)

Respondents reported no difference in level of support for FGC for daughters than daughters-in-law both when asked directly, 7.3% (95% CI [4.6–9.9]) and 8.2% (95% CI [5.4–11.0] respectively, p = 0.645), or indirectly using UCT 19.7% (95% CI [11.9–27.6]) and 25% (95% CI [16.5–33.5], respectively, p = 0.371); see Table 1 and Fig 2. There is, however, evidence of concealment of FGC support (i.e. greater difference between direct and indirect UCT estimates) for both categories of female relatives. When considering hypothetical daughters, 7.3% (95% CI [4.6–9.9]) of respondents were supportive when asked directly, rising to 19.7% (95% CI [11.9–27.6]) when questioned indirectly (p = 0.003). When considering hypothetical daughters-in-law, direct and UCT responses were 8.2% (95% CI [5.4–11.0]) and 25% (95% CI [16.5–33.5]) respectively (p<0.001).

### Individual characteristics of respondents

In addition within the community certain kinds of individual were more likely to hide their views in favour of FGC when questioned directly. We tested whether education, gender or age influenced reported acceptance of FGC, and these results are reported below. The dependent variable in the following paragraphs is the overall proportion of supportive responses regarding FGC, derived from the responses to questions regarding daughters and in-laws combined (unless otherwise stated). Table 1 provides a breakdown of the estimates according to question methodology (direct vs. UCT), individual traits (e.g. gender) and target female (daughter vs daughter-in-law). S1 Table provides more detailed breakdown of the sub group analyses, to explore the relationship between variables, between methodology, and each of the three individual traits (gender, age group and educational level).

#### Gender

**Fig 3:**
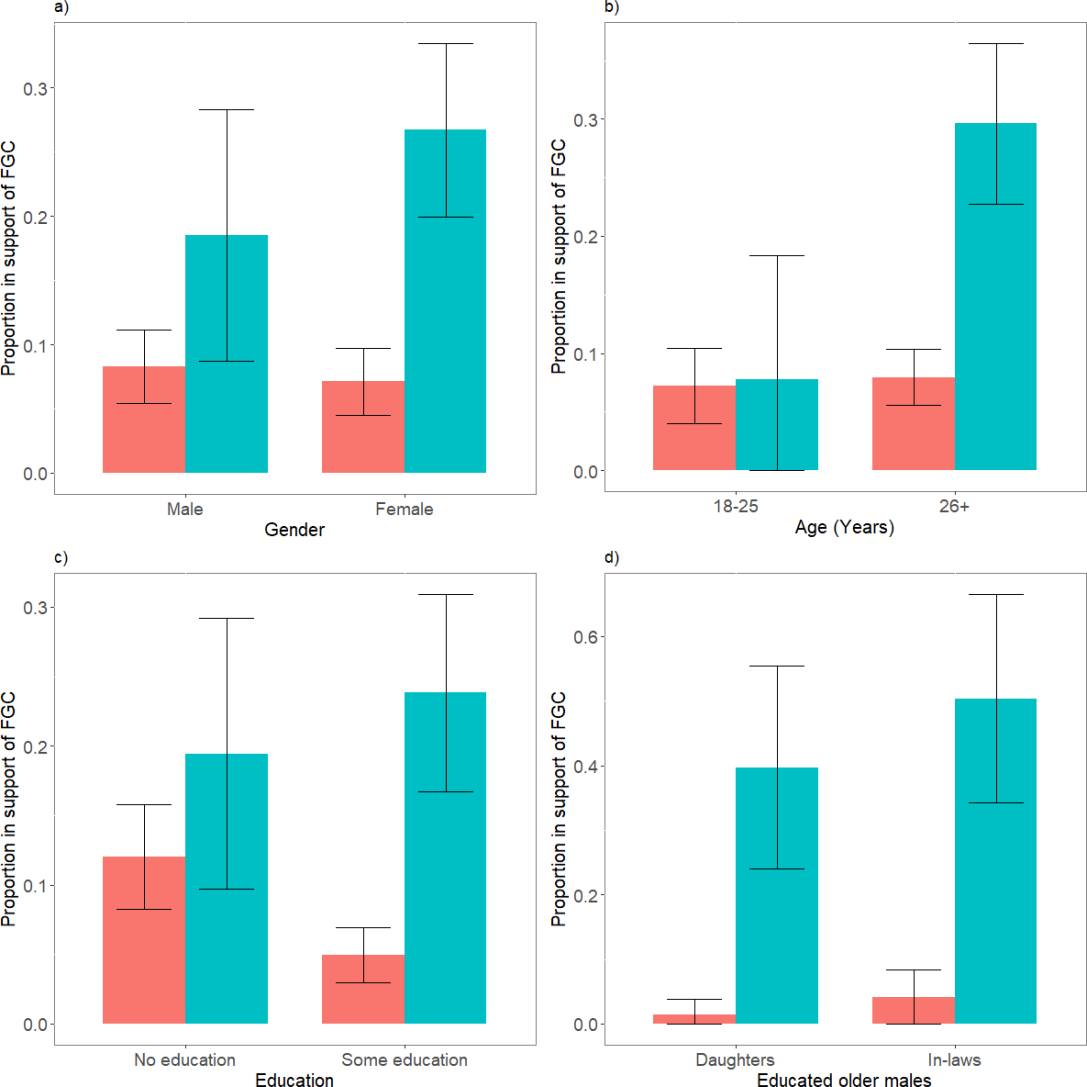
Bar chart comparisons of the proportion of people supporting FGC, using DQ and UCT responses by **a)** gender, **b)** age group and **c)** education level [includes mean estimates of daughter and in-laws combined, n = 1620]. Graph **d)** includes only the subgroup educated, older males [n = 408], with separate estimates for daughters and daughters-in-law. DQ = orange bars, UCT = green bars. The error bars represent confidence intervals at 95%.

Men and women reported similar and low levels of support for FGC when asked directly (8.3% and 7.1%, 95% CIs [5.4–11.1] and [4.5–9.8], p = 0.563; see Fig 3A). Using UCT, women appeared privately more supportive of the practice than men (men: 18.5%, women: 26.7%, 95% CIs [8.7–28.3] vs [20.0–33.5]), but this difference was not significant, (p = 0.178). A comparison of direct and UCT estimates indicates that both men and women concealed their true support for FGC to some degree when questioned directly. Men reported low levels of support for FGC in response to the direct question, 8.3% (95% CI [5.4–11.1]) rising to 18.5% (95% CI [8.7–28.3]) in response to the UCT, (at borderline significance, p = 0.051). A breakdown of these estimates according to target women (presented in Table 1), however suggests that males were less likely to conceal support for FGC in daughters (p = 0.356) than daughters-in-law (although not quite at 5% significance level, p = 0.064). For women DQ reveals that FGC support was 7.1% (95% CI [4.5–9.8]), rising to 26.7% (95% CI [20.0–33.5]) using UCT, (p<0.001), with concealment evident for both daughters and daughters-in-law (see Table 1).

#### Age

When asked directly, individuals in the two age-groups (<26, > = 26) reported similar, low support for FGC (7.2%, 8% respectively; 95% CIs [4.0–10.4], [5.6–10.4], p = 0.716). Indirect estimates, however, indicate that private support was higher among those aged over 26 (29.6%, 95% CI [22.8–36.5]) than those aged <26 (7.8%, 95% CI [0–18.4]; with p<0.001). There also is a significant discrepancy between DQ and UCT estimates of support among the oldest subgroup (> = 26 years), 8% (95% CI [5.6–10.4]) and 29.6% (95% CI [22.8–36.5]) respectively, (p<0.001), but not in the youngest subgroup (<26 years), 7.2% (95% CI [4–10.4]) and 7.8% (95% CI [0–18.4]) respectively, (p = 0.923). (See Fig 3B). These results indicate that older individuals were privately more supportive of FGC than younger individuals, but were also more likely to conceal this support when asked directly.

#### Educational level

When asked directly, uneducated respondents were more likely to admit support for FGC than those who had received formal education (ever attended school) (12.0% compared to 5.0%, 95% CIs [8.2–15.8] vs [2.9–7.0], p = 0.001). UCT, however, suggested a reversal of this with uneducated respondents privately being *less* supportive of FGC than educated individuals (19.4% vs 23.8%, 95% CIs [9.7–29.2] vs [16.7–30.9]); however these differences were not statistically significant (p = 0.476). Educated respondents were more likely to admit their support for FGC when questioned indirectly using UCT than using a direct method; 5.0% expressed direct support for FGC, rising to 23.8% using UCT (95% CIs [2.9–7.0]) vs [16.7–30.9]; p<0.001) (see Fig 3C). For respondents with no schooling, similar biases were not evident; percentage support was 12% using direct questions (95% CI [8.2–15.8]) and 19.4% for UCT (95% CI [9.7–29.2]); and the difference between direct and UCT responses was not significant (p = 0.165). These results indicate that educated and non-educated people did not differ in their privately-held views on the desirability of FGC; however, educated respondents were more likely conceal this support when questioned directly by an interviewer.

### Further sub-group analysis

Prior research suggests that FGC practices may persist in certain subsections of society in which they are normative [16]. We therefore tested whether private acceptance of FGC existed at levels around 50% in any sub-group of the Arsi Oromo population. However, it is worth noting that for this population there are small sample sizes in certain sub-groups, (e.g. there are very few older, educated Arsi Oromo females, or uneducated young adults) meaning that many differences are not statistically significant. Our analyses reveal that high levels of private support for FGC are found among older, educated males (+26 years), where estimated acceptance levels reached 45% (39.7% for daughters, and 50.4% for daughters-in-law; 95% CIs [24–55.4] and [34.2–66.5] respectively. This category of individuals was also the least likely to openly admit a preference for FGC (1.4% and 4.1%; 95% CIs [0–3.9] and [0–8.4]); which is reflected in a large discrepancy between directly-expressed and privately-held views, the largest of any subgroup of individuals within this population (both p<0.001). (See Fig 3D). S1 Table includes a breakdown of sub-group analyses, contrasts between question methodology (DQ vs UCT) and each of the respondent’s individual traits (gender, age and education level).

## Discussion

Here we demonstrate that traditional direct methods, which rely on direct, face-to-face questioning to determine levels of support for FGC are highly unreliable. Comparing direct and indirect response methods in rural Oromia, South Central Ethiopia, we identify substantial underreporting of support for FGC using direct questioning methods. Across the community, privately-held views in favour of FGC are approximately three times higher than those admitted when asked directly by an interviewer. We identify that older individuals hold the strongest views in favour of FGC, but are also the most likely to hide their ‘true’ support for the practice when questioned directly. The lowest concealed support for FGC is among the youngest cohort (<26 year olds) which could suggest that social norms favouring FGC are shifting across the whole community overtime or alternatively that individuals become more accepting of the practice with age. Repeated surveys in this community may help to identify the extent to which either or both of these scenarios are true.

Our results also indicate that educated Arsi Oromo give more socially desirable answers than those individuals without schooling, hiding their ‘true’ FGC intentions when questioned directly. Similar associations linking education and under-reporting of sensitive attitudes (e.g. racist beliefs) have been documented in high-income populations [32], but this link has been harder to establish in low-income settings [14]. The knowledge that the most educated people in the community are inclined to conceal their private views in favour of FGC is highly relevant for public health policy seeking to eradicate the practice. Improved community education on the health risks has long been a major focus for policy. However, to date broad public health information campaigns have had mixed results, and for the most part have not motivated mass abandonment of cutting (see review by Shell-Duncan [15]).

It is however worth noting, that educational attainment for those in our sample attending school is very low (< 3years completed education), and it may be that for acceptance of FGC to decline, much higher levels of education maybe required (e.g. secondary schooling). Lower acceptance of FGC has been identified among the educated Afar pastoralists in Northern Ethiopia using similar UCT methods [14], which may reflect greater schooling and income-generating opportunities available in this population. Among our sample of Arsi Oromo, women’s economic security is very strongly linked to securing a good marriage to a wealthy man, arranged on their behalf by their parents and community elders. In this context, the perceived socio-economic advantages with increased education may actually maintain (or even increase) leverage for educated individuals to “demand” FGC in potential spouses/in laws. Our results suggest that educational expansion (at least in its early stages) may not be enough to change FGC norms of an entire community, rather it may simply heighten secrecy. Community-level intervention schemes seeking to promote the abandonment of FGC, have similarly been linked with under-reporting [14], and change in practices to prevent detection, e.g. cutting at an earlier age [33] in other populations.

Some FGC eradication efforts have sought to target key individuals within communities, many with an emphasis on women, who are considered to be ‘at the forefront of the perpetuation of FGC’. In this Arsi Oromo community and many others where FGC is common, women lead the rites surrounding the practice, and carry out the cutting. Men are often considered to be less directly involved in the process and (when asked directly) in favour of the abandonment of the practice [34]. Our results reveal that both men and women are equally supportive of FGC in our sample, and attempt to conceal their support in front of interviewers. This finding casts doubt on the potential efficacy of FGC eradication programmes with an exclusive focus on women (in this and similar communities).

We find no clear evidence for weaker support for FGC for daughters over daughters-in-law, in line with an evolutionary prediction that parents will be more concerned with controlling the sexual behaviour of their daughters-in-law. Rather, our results support the proposal that the fitness costs and benefits of cutting, in terms of health risks and paternity certainty respectively, are equivalent for daughter and daughters-in-law. Put simply, parents don’t want their sons raising other men’s children, but they also want potential spouses (and future in-laws) to have faith in the fidelity of their daughters. In our community, cutting daughters remains the best way of ensuring good marriages, but also in-law support for daughters when leaving their natal home after marriage [16]. We do, however, find an indication that men may be less inclined to conceal their support for cutting daughters, than for daughters-in-law (although at borderline significance, p = 0.064). Why this should be is currently unclear, but we speculate that this may reflect particular pressures for fathers to openly signal sexual fidelity and hence marriageability of their daughters to potential spouses and in-laws. The suggestion of variation in desirability of FGC based on sex and relatedness supports the evolutionary proposal that sexual conflict in humans is not constant, but may vary across socio-ecological circumstances [19], as well as highlighting a need for further FGC studies exploring the role of kinship and differential kin support.

Finally, our results suggest that it is elders, particularly educated men who hold some of the strongest views in favour of the practice (>45% privately endorse FGC, but these views are hidden when asked directly). This group represents around 12% of the total population, and hold positions of authority in the community, taking on responsibility for village leadership, defence, and key social rites (e.g. arranging marriages). Concealed support and pressure to continue FGC from this powerful and influential group of elders could explain the stubborn persistence of the practice in this and similar communities. For policy-makers, the identification of such sub-groups is important, because individuals are likely to form alliances on the basis of similarity, and because evidence suggests that conformity to normative cultural practices may explain the popularity both of FGC and many other cultural traits [16, 35]. The existence of “pockets” of high support may therefore explain the persistence of FGC in populations in which it is, overall, a minority practice. It is worth noting, however, that the results presented here (and in similar studies) should be interpreted with caution, as there is a statistical requirement for large sample sizes when conducting unmatched count technique analyses [11]. Replication of our methods in larger samples would allow “high support” subgroups to be confidently identified, and key subgroups targeted for interventions in this and in other populations.

## Conclusion

Our results demonstrate the inadequacy of traditional, yet widely used, direct questioning methods, and the potential for indirect questioning techniques to improve understanding of culturally-sensitive topics, like FGC. Comparing direct and indirect methods we reveal how some individuals (particularly influential older people) are more inclined to hide their “true” support for female genital cutting in rural Ethiopian community. While there is a requirement for large sample sizes to use these techniques, our findings support a growing view that indirect questioning methods can be usefully applied to improve understanding on a range of sensitive health-related behaviours, as well as to improve reliability in monitoring and evaluation of health intervention initiatives across low-income settings.

## Supporting information

S1 Fig Sensitive questions in four versions of the survey. (DOCX)

S2 Fig Generating the list, data quality tests and sampling strategy. (DOCX)

S1 Table Sub group analyses between question methodology (direct versus unmatched count technique) and individual traits (gender, education level and age group), with combined estimates for FGC support (for both daughters and daughters in law), n = 1620. (DOCX)

S1 Dataset (SAV)
