# Supplementary material for: Indirect questioning method reveals hidden support for female genital cutting in South Central Ethiopia
Source: PLoS One. 2018 May 2;13(5):e0193985. doi: 10.1371/journal.pone.0193985 (PMC5931472; doi:10.1371/journal.pone.0193985)
Supplement: S1 Fig — (DOCX) [file pone.0193985.s001.docx]

**S1 Fig.**

***I’d like you to imagine that you are the parent of a girl who has not yet become an adult. Family have wishes and aims for their children. There are some things that they hope will happen to them by the time they are adults, or which they will try to ensure happens if possible. There are other things that they do not particularly want or do not care about.***

***Here I have some cards. On each card is written something that you might (or might not) want for your daughter by the time she is an adult.***

- If using survey 1A or 1B, read the following:

***In a moment I’ll read out each card, all you have to do is tell me whether you would want this for a daughter.***

1A:

| Would you want this for your daughter?  (1=Yes, 0=No) | | | |
| --- | --- | --- | --- |
| Early marriage | Work in the city | Go to college | Live close to home |
|  |  |  |  |

1B:

| Would you want this for your daughter?  (1=Yes, 0=No) | | | | |
| --- | --- | --- | --- | --- |
| Early marriage | Work in the city | Go to college | FGC | Live close to home |
|  |  |  |  |  |

- If using surveys 2A or 2B, read the following:

***We want to know about peoples’ views about being a parent, but we also want them to be able to keep their views private so that we get honest answers. So please don’t tell me which of the things on these cards you personally would want for your daughter. Instead, I’d like you to tell me how many of these cards show things that you want for your daughter.***

***It’s important that you don’t tell me which individual things you are choosing, just give me a number. You can choose as many or as few as you like. If you’d like to hold or move the cards that is fine, but please don’t tell me which particular cards you are choosing.***

***OK: how many of these would you want for your daughter?***

2A:

| Would you want this for your **daughter**?  (1=Yes, 0=No) | | | |
| --- | --- | --- | --- |
| Early marriage | Work in the city | Go to college | Live close to home |
|  |  |  |  |

2B:

| 1B: Would you want this for your **daughter**?  (1=Yes, 0=No) | | | | |
| --- | --- | --- | --- | --- |
| Early marriage | Work in the city | Go to college | FGC | To live close to home |
|  |  |  |  |  |

| Daughter: How many cards were selected? | Total number of cards |
| --- | --- |
|  |  |

***OK, we’re going to repeat that a second time. This time, I’d like you to imagine that you are the parent of a man who is soon going to be married. I’m going to read out those things again, and this time, please think about which of these things you would want your son’s wife to have. Your preferences might be the same as before, or a bit different this time. Either of these is fine; please just be honest.***

2A:

| Would you want this for your **son’s wife**?  (1=Yes, 0=No) | | | |
| --- | --- | --- | --- |
| Early marriage | Work in the city | Go to college | Live close to home |
|  |  |  |  |

2B:

| 1B: Would you want this for your **son’s wife**?  (1=Yes, 0=No) | | | | |
| --- | --- | --- | --- | --- |
| Early marriage | Work in the city | Go to college | FGC | To live close to home |
|  |  |  |  |  |

| **Son’s wife**: How many cards were selected? | Total number of cards |
| --- | --- |
|  |  |
