# Supplementary material for: Indirect questioning method reveals hidden support for female genital cutting in South Central Ethiopia
Source: PLoS One. 2018 May 2;13(5):e0193985. doi: 10.1371/journal.pone.0193985 (PMC5931472; doi:10.1371/journal.pone.0193985)
Supplement: S2 Fig — (DOCX) [file pone.0193985.s002.docx]

**S2 Fig.**

**Generating the list.** To generate non-sensitive items for the list, focus groups discussions were conducted with local residents, who were asked to report popular local preferences regarding female relatives’ and in-laws’ traits. These discussions and a free-listing exercise generated an extended list of potential items, from which four were selected for inclusion in the survey. The final four items were chosen so as to minimise the chance of floor and ceiling effects – that is, of participants preferring either all or none of the items. Such effects can be problematic because they effectively reveal the participant’s attitude to the sensitive item [9, 11]. Following practices advocated in prior research to mitigate floor/ceiling effects, [11] four items were selected such that: a) one item was expected to be unpopular (early marriage) b) one item was expected to be popular (go to college) and c) two items were expected to be seen as incompatible (work in the city, and live close to home). Expectations regarding the popularity of different items were confirmed in a piloting stage (n=150), and low levels of floor/ceiling effects were observed in the final dataset. The % of respondents selecting all/none of the cards was <4.5%.

A check for independence of responses was also undertaken to ensure that during direct questioning, adding the sensitive item did not change people’s tendency to respond “yes” to the other four items. This “additional item” test was passed, there was no statistical difference between in responses to direct questions with and without the FGC card (t=1.04, p=0.297).

**Sampling strategy.** Our study was designed to ensure to there were adequate numbers and enough statistical power to perform UCT analyses (indirect questions were 70% of the total, n=1117), while reducing the relative number of responses to direct questions without the FGC card (4 card control group), which was included only to test the quality of the UTC list. Performing power calculations we identified we could achieve 80% power to detect an increase/decrease in the proportion of “yeses” in the direct question control and treatment groups we split the sample as follows: 20% (n=331) answered the direct question with the sensitive item, FGC, [5 item treatment], and 8% (n=177) answered the direct question without the sensitive item [4 item control].

**Sampling method.** All four versions of the survey were randomly assigned across households, and within household by gender and marital status. Each same-sex interviewer received a pile of surveys each morning, which had been randomly sorted by a data editor, including all 4 versions of the survey. Interviewers then travelled house to house, administering surveys to alternate households selected from a village plan supplied by the local district administrators. Accordingly, a random sample of 50% the households in the community were surveyed. Within each household, two surveys were completed by an equal and randomly selected sample of adult male and female, married and unmarried respondents from a household list supplied by the local authorities.
