# Supplementary material for: Indirect questioning method reveals hidden support for female genital cutting in South Central Ethiopia
Source: PLoS One. 2018 May 2;13(5):e0193985. doi: 10.1371/journal.pone.0193985 (PMC5931472; doi:10.1371/journal.pone.0193985)
Supplement: S1 Table — (DOCX) [file pone.0193985.s003.docx]

**S1 Table.**

| Respondent | | | n | Direct estimate (SE) | UCT estimate (SE) | P-values |
| --- | --- | --- | --- | --- | --- | --- |
| male | no education | 18-25 years | 13 | 0.5 (0.340) | 0.667 (0.819) | 0.850 |
| male | no education | 26+ years | 145 | 0.196 (0.051) | 0 (0) | 0.009 |
| male | some education | 18-25 years | 245 | 0.085 (0.024) | 0 (0) | 0.091 |
| male | some education | 26+ years | 408 | 0.027 (0.013) | 0.450 (0.058) | <0.001 |
| female | no education | 18-25 years | 35 | 0 (0) | 0.390 (0.162) | 0.016 |
| female | no education | 26+ years | 388 | 0.1 (0.021) | 0.308 (0.052) | <0.001 |
| female | some education | 18-25 years | 239 | 0.049 (0.023) | 0.199 (0.063) | 0.026 |
| female | some education | 26+ years | 147 | 0.036 (0.024) | 0.246 (0.076) | 0.008 |
